# Late Periprosthetic Joint Infection due to *Staphylococcus lugdunensis* Identified by Matrix-Assisted Laser Desorption/Ionisation Time of Flight Mass Spectrometry: A Case Report and Review of the Literature

**DOI:** 10.1155/2011/608919

**Published:** 2011-07-12

**Authors:** Florian Szabados, Agnes Anders, Martin Kaase, Lennart Marlinghaus, Sören G. Gatermann, Wolfram Teske, Thomas Lichtinger

**Affiliations:** ^1^Institute for Hygiene and Microbiology, Department of Medical Microbiology, Ruhr-University Bochum, 44801 Bochum, Germany; ^2^Department for Orthopaedic Surgery, Ruhr-University Bochum, 44791 Bochum, Germany

## Abstract

*Staphylococcus lugdunensis*, member to the group of coagulase-negative staphylococci, is previously thought to be rarely isolated. Recently other staphylococci have been described, which were supposedly related to *S. lugdunensis*, such as *Staphylococcus pseudolugdunensis* and *Staphylococcus pettenkoferi*. To decrease the rate misidentifications, an accurate identification method, such as matrix-assisted laser desorption ionization time of flight mass spectrometry or molecular methods, should be used. *S. lugdunensis* is usually associated with severe infections similar to those caused by *S. aureus*. Moreover, it has been described that skin infections due to *S. lugdunensis* are severely underreported and could be also underreported in periprosthetic joint infections. Ours is the first case of a late periprosthetic infection of the hip due to *S. lugdunensis*, identified by matrix-assisted laser desorption/ionisation time-of-flight mass spectrometry. A periprosthetic infection due to *S. lugdunensis* should be treated according to protocols of *S. aureus* periprosthetic infections, and therefore an accurate species identification is desirable.

## 1. Introduction

*Staphylococcus lugdunensis *has been described as a rare pathogen, which causes diseases similar to those caused by *Staphylococcus aureus *[1]. The reaction with pyrrolidonyl arylamidase (PYR) and ornithine decarboxylase (ODC) has been described to distinguish *S. lugdunensis *from other staphylococci [2]. Nevertheless, a PYR-negative *S. lugdunensis* isolate has been recently described [3], and it is likely that also ODC-negative *S. lugdunensis* exits. Moreover, species supposedly related to *S. lugdunensis*, such as *Staphylococcus pseudolugdunensis *[4] and *Staphylococcus pettenkoferi *[5, 6], have been described. *S. lugdunensis*, identified by superior methods, such as the recently described matrix-assisted laser desorption/ionisation time-of-flight mass spectrometry-(MALDI TOF MS-) based identification [7], could be used to redefine the species of *S. lugdunensis* and newly investigate their prevalence and pathogenicity.

## 2. Case Presentation

A 47-year-old male patient was hospitalized because of severe pain and swelling of the right leg. 

**Figure 1:**
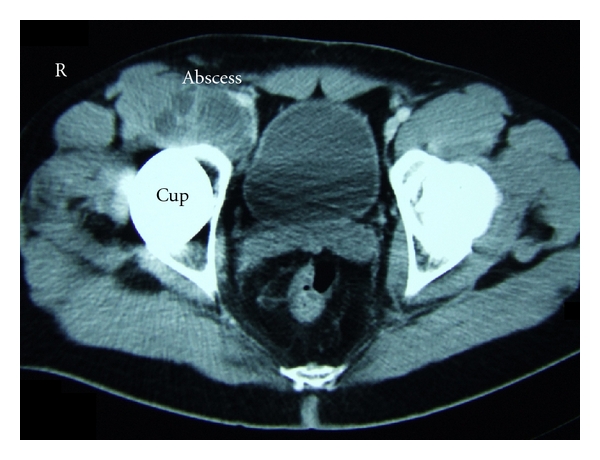
Abscess in front of the hip arthroplasty due to *S. lugdunensis.* The computer tomography (CT) shows a large cystic formation ventral to the hip reaching the psoas-muscle.

A cementless total hip arthroplasty was implanted two and a half years before, due to a previous osteonecrosis of the femoral head. In addition, the patient suffered from noninsulin-dependent diabetes mellitus type 2 and hepatitis B. The computer tomography (CT) showed a large cystic formation ventral to the hip reaching the psoas muscle (Figure 1). The CRP was elevated 23-fold (CRP = 116 mg/L ) and in the puncture *S. lugdunensis* was cultured from several samples. CT-assisted drain was placed into the cyst and left for several days. A targeted antibiotic therapy with oxacillin (2 g every 8 h) and rifampicin (600 mg every 12 h) was intravenously started and switched to oral application and maintained for two months. During the antibiotic therapy the cystic lesion was decreasing.

Two months later the patient complained again about pain in the right hip. Plain radiographs showed no signs of loosening of the cup or the femoral stem. The CRP was elevated with 76 mg/L. A skeletal scintigram was performed. The enhancement of the synovia in the blood-pool phase and the increase of bone metabolism in the late phase of the bone scintigraphy were interpreted as a sign of a prosthetic infection. In the puncture of the joint itself *S. lugdunensis *was cultured as the causative pathogen again. In summary these results showed now a periprosthetic infection of the hip joint.

Therefore a two-staged revision of the prosthesis was performed. In the first stage, the prosthesis was explanted and replaced by a gentamicin-containing bone cement spacer into the acetabulum and a gentamicin-containing sponge into the femur. In an intraoperatively taken wound swab *S. lugdunensis* was cultured again. An antibiotic therapy was started with doxycycline (100 mg every 12 h) and rifampicin (600 mg every 12 h) and maintained for six weeks. 

In the second stage four months later a conventional hip prosthesis was successfully reimplanted and doxycycline (100 mg every 12 h) and rifampicin (600 mg every 12 h) were administered for two weeks again. Another four weeks later the prosthetic hip joint was revised because of recurrent dislocations of the head out of the cup. No bacteria were cultured from several intraoperatively taken wound swabs nor significant elevations of the CRP were observed. In a clinical follow-up 15 months later the patient showed a fair function of the hip. Plain radiographs and blood test results also showed no evidence of a periprosthetic reinfection.

### 2.1. Species Identification in Our Case

**Figure 2:**
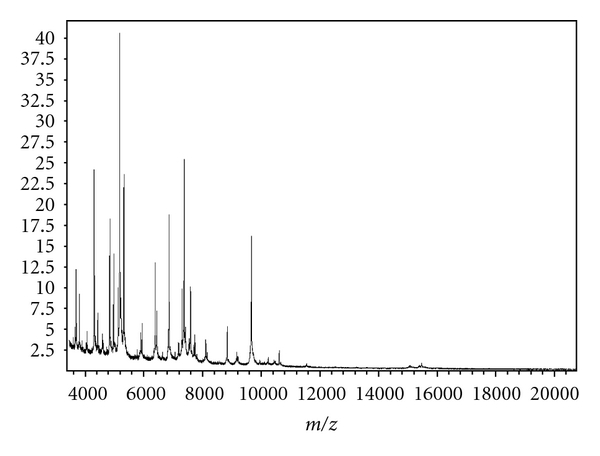
*S. lugdunensis* whole cell matrix-assisted laser desorption/ionisation time-of-flight MS fingerprint. Bacteria were covered with a layer of matrix (*α*-Cyano-4-hydroxy-cinnamic acid) and submitted for analysis. The proteomic fingerprint in the mass-to-charge ratio (Da) from 3000 Da to 15.000 Da was species specific and was matched to the Biotyper 2.0 database (Bruker Daltonics). Within a few minutes a highly accurate identification up to the species level was reported.

Staphylococci were primarily identified by typical colony morphology and odor and were suspected as *S. lugdunensis*. Staphylococci were then misidentified by the GPI card by the Vitek-2 automated identification system (bioMérieux, Marcy l'Etoile, France). MALDI-TOF MS identified these staphylococci as *S. lugdunensis *(Figure 2). The species was confirmed by amplification of the species-specific *tanA* and *fbl* gene as previously described [7–9]. In vitro susceptibility testing was performed by the AST-580 card by the Vitek-2 automated identification system (bioMérieux). An additional PCR was used to rule out the presence of a *mecA* gene.

## 3. Discussion

### 3.1. Importance of Accurate Species Identification in Periprosthetic Infections

In the few published literature concerning *S. lugdunensis* caused prosthetic infections mostly knee and other prosthetic sites rather than hip arthroplasties were included [1, 10]. Up to now, data on periprosthetic hip infection due to *S. lugdunensis* is scarce. Within the group of CoNS, *S. epidermidis* has been reported to be an important pathogen in prosthetic joint infections. The contribution as a causative pathogen has been also discussed controversially [11], since *S. lugdunensis* is thought to be part of the normal skin flora, isolated primarily from lower abdomen and extremities [12]. An *ica*-dependent and an *ica*-independentbiofilms havebeen described, and these biofilms were discussed as a pathogenicity factor in biomaterial-associated infections [13]. Unexpectedly, data on pathogenicity factors of *S. lugdunensis* is scarce. Only a few reports have been described with regard to fibrinogen-binding adhesins [14, 15] and an *S. lugdunensis* synergistic hemolysin (SLUSH). The genome of a clinical strain of *S. lugdunensis* has been newly published and reveals a variety of pathogenicity factors such as the presence of a toxin pathogenicity island and further putative adhesins [16]. Notably, only 28% of the *S. lugdunensis* strains bind to fibrinogen [3], indicating differences between *S. lugdunensis* isolates with regard to binding to extracellular matrix proteins and their supposed pathogenicity. Since a long time *S. lugdunensis* is thought to be a rare but significant pathogen [1], but recently it was reported that skin and soft tissue infections due to *S. lugdunensis* are often underreported [17]. Typical morphological characteristics of this bacterium, such as a characteristic odor and a strong hemolysis after 48 h of incubation, have been described, which could help to increase the identification of *S. lugdunensis* [17]. In addition, the reaction with pyrrolidonyl arylamidase (PYR) and ornithine decarboxylase (ODC) has been described to distinguish *S. lugdunensis *from other staphylococci [2]. In contrast to this, a PYR-negative *S. lugdunensis* isolate has recently been reported [3], indicating that a significant amount of stains will be misidentified using the latter strategy. An ODC-negative *S. lugdunensis* has not been reported yet, but it is likely that such isolates also exit. Since *S. lugdunensis* usually is confirmed by a positive ODC reaction, previously identified collections likely do not contain an ODC-negative isolate per enclosure definition. Moreover, supposedly related new species have been described, such as *S. pettenkoferi* [5, 6] and *S. pseudolugdunensis *[4]. Some isolates of *S. lugdunensis* could be misidentified as other coagulase-negative staphylococci, especially when only a biochemical identification was performed.

Prosthetic joint infections can be classified in early infections, delayed infections, and late infections [18]. Early infections occur within three to four weeks. Delayed infections were defined as those up to 12 months, and late infections were defined as those occurring after more than 12 months [19]. Early and delayed infections are thought to be associated with pathogens introduced at the time of surgery, whereas late infections are discussed to be haematogenously acquired [19]. Staphylococci are most frequently isolated. For instance, coagulase negative staphylococci account for about 30% of infections in knee arthroplasty [20]. *S. aureus *is the most common haematogenously transmitted pathogen in all periprosthetic infections [19]. In many clinical microbiological laboratories coagulase negative staphylococci were identified by basic biochemical methods or supposedly specific reactions, such as PYR and ODC. A presumptive species identification using such methods is suitable for many staphylococcal species, but with an accuracy below of that of MALDI TOF MS and other molecular methods [3, 21]. This lower accuracy, depending on the sample type, is usually believed to be sufficient. In case of prosthetic or periprosthetic infections due to suspected *S. lugdunensis,* these isolates should be confirmed by molecular methods [8, 9, 22] or by MALDI TOF MS, which has been described as an easy-to-handle, fast, and reliable method for the identification of staphylococci [7, 21, 23].

### 3.2. Microbiological Sampling and Surgical Management

One of the most important tests in the evaluation of a potential periprosthetic infection is culture of aspirated fluids and tissue samples. The positive predictive value of microbiologic culture is low, when performed in all patients before revision total hip arthroplasty, even when the clinical features did not necessarily suggest infection [24]. In a later study the positive predictive value of microbiologic culture was significantly higher in a collection of knee arthroplasties. This difference was discussed as a potential difference between knee and hip arthroplasties. On the other hand, the prevalence of infection in the second study was clearly higher (29%) compared to the first study with 2%. This also indicates that the positive predictive value of microbiologic culture is low, if solely used as a screening test for infection instead of as a confirmatory test for patients in whom clinical findings have raised the suspicion of infection [25]. When the culture results were correlated to the erythrocyte sedimentation rate or CRP, the sensitivity could be also increased [26]. In case of suspected periprosthetic infections it is recommended that five distinct intraoperative samples should be taken with separate instruments. An indistinguishable organism from at least three samples is strongly associated with infection. If a periprosthetic infection of the hip is diagnosed, the surgeon has to choose the optimal therapeutic strategy for the individual patient. For instance, limited surgical management involved debridement of a joint with exchange of modular components but retaining the prosthesis itself, combined with prolonged antibiotic therapy [19]. Prosthetic infections due to *S. aureus* seem to be associated with a higher rate of failure, when a limited strategy was compared to revision arthroplasty [27]. *S. lugdunensis* is often associated with severe clinical diseases similar to infections caused by *S. aureus* [1]. Therefore we performed a staged revision hip arthroplasty and not a limited surgical procedure in the reported case. Up to now, the treatment was successful, and there are no signs of a reinfection of the hip.

## 4. Conclusion

Periprosthetic hip infections due to *S. lugdunensis* are rarely reported. Depending on the identifying algorithm used in diagnostic laboratories, *S. lugdunensis* could be underreported or misidentified as other coagulase negative staphylococci. Identification of staphylococci using MALDI-TOF MS is straightforward, and the identification accuracy is equivalent to molecular methods. Therefore, these methods should be used for species identification of coagulase negative staphylococci rather than a previously described biochemical identification. *S. lugdunensis* is an important and often underestimated pathogen in severe skin and soft tissue infection, therefore, it seems to be likely that this pathogen is also underestimated in prosthetic and periprosthetic infections of the hip. Periprosthetic hip infections due to *S. lugdunensis* should be investigated in further detail to gain insights into the pathogenicity of this outstanding pathogen.

##  Conflict of Interests

The authors certify that there is no actual or potential conflict in relation to this paper.

## References

[1] Frank KL, Del Pozo JL, Patel R (2008). From clinical microbiology to infection pathogenesis: how daring to be different works for *Staphylococcus lugdunensis*. *Clinical Microbiology Reviews*.

[2] Tan TY, Ng SY, He J (2008). Microbiological characteristics, presumptive identification, and antibiotic susceptibilities of *Staphylococcus lugdunensis*. *Journal of Clinical Microbiology*.

[3] Szabados F, Nowotny Y, Marlinghaus L (2011). Occurrence of genes of putative fibrinogen binding proteins and hemolysins, as well as of their phenotypic correlates in isolates of *S. lugdunensis* of different origins. *BMC Research Notes*.

[4] Tang YW, Han J, McCormac MA, Li H, Stratton CW (2008). *Staphylococcus pseudolugdunensis* sp. nov., a pyrrolidonyl arylamidase/ornithine decarboxylase-positive bacterium isolated from blood cultures. *Diagnostic Microbiology and Infectious Disease*.

[5] Trulzsch K, Grabein B, Schumann P (2007). *Staphylococcus pettenkoferi* sp. nov., a novel coagulase-negative staphylococcal species isolated from human clinical specimens. *International Journal of Systematic and Evolutionary Microbiology*.

[6] Loïez C, Wallet F, Pischedda P (2007). First case of osteomyelitis caused by ‘*Staphylococcus pettenkoferi*’. *Journal of Clinical Microbiology*.

[7] Szabados F, Woloszyn J, Richter C, Kaase M, Gatermann S (2010). Identification of molecularly defined *Staphylococcus aureus* strains using matrix-assisted laser desorption/ionization time of flight mass spectrometry and the Biotyper 2.0 database. *Journal of Medical Microbiology*.

[8] Noguchi N, Goto K, Ro T (2009). Using the tannase gene to rapidly and simply identify *Staphylococcus lugdunensis*. *Diagnostic Microbiology and Infectious Disease*.

[9] Pereira EM, Oliveira FL, Schuenck RP, Zoletti GO, Dos Santos KR (2010). Detection of *Staphylococcus lugdunensis* by a new species-specific PCR based on the fbl gene. *FEMS Immunology and Medical Microbiology*.

[10] Shah NB, Osmon DR, Fadel H (2010). Laboratory and clinical characteristics of *Staphylococcus lugdunensis* prosthetic joint infections. *Journal of Clinical Microbiology*.

[11] Hubiche T, Del Giudice P, Roudière L (2009). *Staphylococcus lugdunensis* in skin infections: pathogen or colonizing bacterium?. *Journal of Clinical Microbiology*.

[12] Bieber L, Kahlmeter G (2010). *Staphylococcus lugdunensis* in several niches of the normal skin flora. *Clinical Microbiology and Infection*.

[13] Mack D, Fischer W, Krokotsch A (1996). The intercellular adhesin involved in biofilm accumulation of *Staphylococcus epidermidis* is a linear beta-1,6-linked glucosaminoglycan: purification and structural analysis. *Journal of Bacteriology*.

[14] Geoghegan JA, Ganesh VK, Smeds E, Liang X, Höök M, Foster TJ (2010). Molecular characterization of the interaction of staphylococcal microbial surface components recognizing adhesive matrix molecules (MSCRAMM) ClfA and Fbl with fibrinogen. *Journal of Biological Chemistry*.

[15] Nilsson M, Bjerketorp J, Guss B, Frykberg L (2004). A fibrinogen-binding protein of *Staphylococcus lugdunensis*. *FEMS Microbiology Letters*.

[16] Tse H, Tsoi HW, Leung SP, Lau SK, Woo PC, Yuen KY (2010). Complete genome sequence of *Staphylococcus lugdunensis* strain HKU09-01. *Journal of Bacteriology*.

[17] Bocher S, Tonning B, Skov RL, Prag J (2009). *Staphylococcus lugdunensis*, a common cause of skin and soft tissue infections in the community. *Journal of Clinical Microbiology*.

[18] Trampuz A, Zimmerli W (2005). Prosthetic joint infections: update in diagnosis and treatment. *Swiss Medical Weekly*.

[19] Moran E, Byren I, Atkins BL (2010). The diagnosis and management of prosthetic joint infections. *Journal of Antimicrobial Chemotherapy*.

[20] Stefánsdóttir A, Johansson D, Knutson K, Lidgren L, Robertsson O (2009). Microbiology of the infected knee arthroplasty: report from the Swedish Knee Arthroplasty Register on 426 surgically revised cases. *Scandinavian Journal of Infectious Diseases*.

[21] Dupont C, Sivadon-Tardy V, Bille E (2010). Identification of clinical coagulase-negative staphylococci, isolated in microbiology laboratories, by matrix-assisted laser desorption/ionization-time of flight mass spectrometry and two automated systems. *Clinical Microbiology and Infection*.

[22] Chatzigeorgiou KS, Siafakas N, Petinaki E, Zerva L (2010). fbl gene as a species-specific target for *Staphylococcus lugdunensis* identification. *Journal of Clinical Laboratory Analysis*.

[23] Harris LG, El-Bouri K, Johnston S (2010). Rapid identification of staphylococci from prosthetic joint infections using MALDI-TOF mass-spectrometry. *International Journal of Artificial Organs*.

[24] Barrack RL, Harris WH (1993). The value of aspiration of the hip joint before revision total hip arthroplasty. *Journal of Bone and Joint Surgery—Series A*.

[25] Bauer TW, Parvizi J, Kobayashi N, Krebs VE (2006). Diagnosis of periprosthetic infection. *Journal of Bone and Joint Surgery—Series A*.

[26] Spangehl MJ, Masri BA, O’Connell JX, Duncan CP (1999). Prospective analysis of preoperative and intraoperative investigations for the diagnosis of infection at the sites of two hundred and two revision total hip arthroplasties. *Journal of Bone and Joint Surgery—Series A*.

[27] Byren I, Bejon P, Atkins BL (2009). One hundred and twelve infected arthroplasties treated with “DAIR” (debridement, antibiotics and implant retention): antibiotic duration and outcome. *Journal of Antimicrobial Chemotherapy*.

